# Therapeutic efficacy and safety of home-based portable laser irradiation on patients with wrist pain: a single-blinded randomized controlled trial

**DOI:** 10.1007/s10103-024-03975-7

**Published:** 2024-01-27

**Authors:** Young-Ji Yun, Da-Sol Kim, Yu Hui Won, Sung-Hee Park, Myoung-Hwan Ko, Jeong-Hwan Seo, Gi-Wook Kim

**Affiliations:** 1 Department of Physical Medicine and Rehabilitation, Jeonbuk National University Medical School, Jeonju, Republic of Korea; 2 Research Institute of Clinical Medicine of Jeonbuk National University - Biomedical Research Institute of Jeonbuk National University Hospital, Jeonju, Republic of Korea

**Keywords:** Laser therapy, Wrist, Pain, Quality of life, Physical therapy modality, Photobiomodulation

## Abstract

The purpose of this study is to confirm the effect of small, portable low-level laser therapy (light sources in square configuration: 830 nm GaAs diode 3.2 mW at the center, 4 × 650 nm InGaAIP diodes over the corners) treatment in reducing and enhancing hand function in patients with wrist pain. This study was a prospective, randomized, sham-controlled, and home-based self-therapy trial. A total of thirty subjects with wrist pain were enrolled. All participants received low-level laser therapy on painful area at the wrist. The experimental group (*n* = 15) received laser stimulation, while the control group (*n* = 15) received sham stimulation using identical equipment that generated only a red light without the laser output. Both groups self-treated for 30 min a day, 5 days per week for 3 weeks, total of 15 sessions. The primary outcome was assessed using a visual analogue scale (VAS) for wrist pain from 0 (painless) to 10 (extreme pain). The secondary outcomes were measured with patient-rated wrist evaluation (PRWE), grip strength, lateral, palmar, and tip pinch strength. Measures were taken before and after treatment. A total of thirty participants provided outcome data. After the intervention, both groups showed a significant decrease in VAS score, from 4.93 to 3.67 in experimental group, from 5.53 to 4.00 in control group (the experiment group: *p* = 0.020, the control group: *p* = 0.003). The experimental group showed a significant improvement in function scale score (*p* = 0.012), the control group did not. Lateral and pinch strength was significantly improved in the experimental group (*p* = 0.017) and in the control group (*p* = 0.034) respectively. There were no side effects in the patients. Medical laser irradiation is a portable and easy-to-use laser irradiator without side effects. Clinical Trial Registration number: KCT0006604.

## Introduction

As the number of people and the time being exposed to computers and smartphones over a sustained period grows, associated side effects are increasingly appearing, including work-related musculoskeletal disorders (WMSDs) especially wrist disease [1, 2]. WMSDs of the wrist are soft tissue disorders of non-traumatic origin that include a wide range of inflammatory and degenerative conditions affecting muscles, tendons, ligaments, joints, and peripheral nerves [3]. WMSDs of the wrist are caused by continuous and repeated movements that result in cumulative injuries to muscles, tendons, or nerves. The prolonged and frequent use of computers and smartphones places considerable load on fingers and wrist joints, causing pain, and over time may result in functional disorders of the wrist [2, 4].

When pain occurs due to sustained overuse, take a break as a general treatment. Treating WMSDs of the wrist first requires that the patient avoid activities that cause the injury, including changing work and lifestyle conditions. Application of hot or cold pack, physical exercises that include stretching, pharmacological interventions such as non-steroidal anti-inflammatory drugs (NSAIDs), and injection therapy also can be used to treat WMSDs of the wrist [5]. Various physical agent modalities, including thermal, mechanical, and electromagnetic stimulation, have been assessed as non-surgical options for pain management, tissue healing, and muscle tone control [6].

Photobiomodulation therapy (PBMT), also known as low-level laser therapy (LLLT), has been used to treat musculoskeletal disorders because it contributes tissue regeneration, promoting angiogenesis, fibroblast proliferation, collagen synthesis, and producing anti-inflammatory effects [7, 8]. This technology is being developed worldwide, and studies are being conducted to verify its effects [9]. PBMT using wavelengths of light from 632 to 904 nm is presently used to treat musculoskeletal disorders [10]. The effectiveness of laser therapy has not yet been a standardized optimal treatment parameter, and there is controversial clear effect of PBMT [11]. Nevertheless, there were some papers that PBMT was an effective treatment option for wrist pain including musculoskeletal disease and carpal tunnel syndrome [12, 13].

Most hospital devices currently employing this treatment generally use large and heavy medical laser irradiator material. Therefore, clinicians in the PBM field claimed the portable home use of PBM device for chronic pain management but studies describing treatments with PBM devices at home for WMSDs of wrist pain are scarce [14]. In this paper, we investigate the therapeutic efficacy of a newly invented home-based portable laser irradiator (Epione™) intended to achieve improvements in hand pain, function, and safety in patients with wrist pain.

## Methods

### Study design

This study was a prospective, randomized, sham-controlled, home-based trial registered at the Clinical Research Information Service, which is under the authority of the Korea Disease Control and Prevention Agency (Registration number: KCT0006604) and was approved by the Institutional Review Board (IRB No. 2019–05-078–013). This study applied the Consolidated Standards of Reporting Trials (CONSORT) guidelines for non-pharmaceutical trials.

This study was conducted at the Rehabilitation Center of OO University Hospital between February 2021 and July 2021. Each participant who fulfilled the inclusion criteria was randomly assigned to one of two groups by a clinical research coordinator (a clinical research nurse not involved in the assessments) and this assignment was concealed from the investigators. They demonstrated the procedure and its potential side effects, obtained signed consent forms, and guided the participants. Study participants were randomized into either the experimental group (Epione™) or the control group. Both groups were treated for a total of 15 sessions: 30 min per day, 5 days per week, over the course of 3 weeks. The experiment group was treated for wrist pain with the medical laser irradiator while the control group received sham stimulation that mimicked its shape and color. In cases of bilateral wrist pain, more severe unilateral wrists were targeted. The clinical research coordinator instructed the patients to keep a daily diary treatment to monitor the home-based self-therapy. Participants were only allowed to take their medication which was started at a month before screening. They were not allowed to change the dose or type of medication or start any other types of treatments for wrist pain during the trial. Patients were assessed twice, first on the day before treatment began (pretreatment) and again within 4 days of the end of treatment (post-treatment). Each patient completed a daily self-therapy checklist to track treatment and record any adverse effects.

### Randomization

Study participants were randomized into either the control or the experimental group and underwent intervention protocols. Simple randomization was done using the random number table of the Randomization.com website. Seed values used at randomization were also recorded. Randomization was completed before the first participant screening, and results were generated by a researcher not involved in study enrollment or intervention. The assessors collecting study data were unaware of group assignments throughout the trial.

### Participants

Study participants were recruited through a notice posted on a bulletin board within the hospital and screened by a rehabilitation physician. Written informed consent was obtained from all participants before randomization. All research was conducted in accordance with the ethical standards set forth in the Declaration of Helsinki. Patients were selected based on the following inclusion criteria: (1) over 20 years of age, (2) moderate wrist pain that interference with daily activity of life, numerical rating score 4–6, (3) able to communicate, (4) capable of following necessary instructions, (5) deemed eligible for participation after examination by the research director. Exclusion criteria included (1) a trauma or operation of the wrist, (2) cases in which the pain was suspected to arise from another origin, such as cervical radiculopathy, other than the wrist, (3) light-sensitive subjects with a history of burn, allergy, or sensitivity in response to sunlight or light therapy, and (4) injection therapy in response to symptoms related to wrist joint pain within the previous month.

All study participants initially complained of moderate wrist pain (mean VAS 4.95–5.53), which implied mild to moderate interference with functioning.

### Intervention

**Fig. 1:**
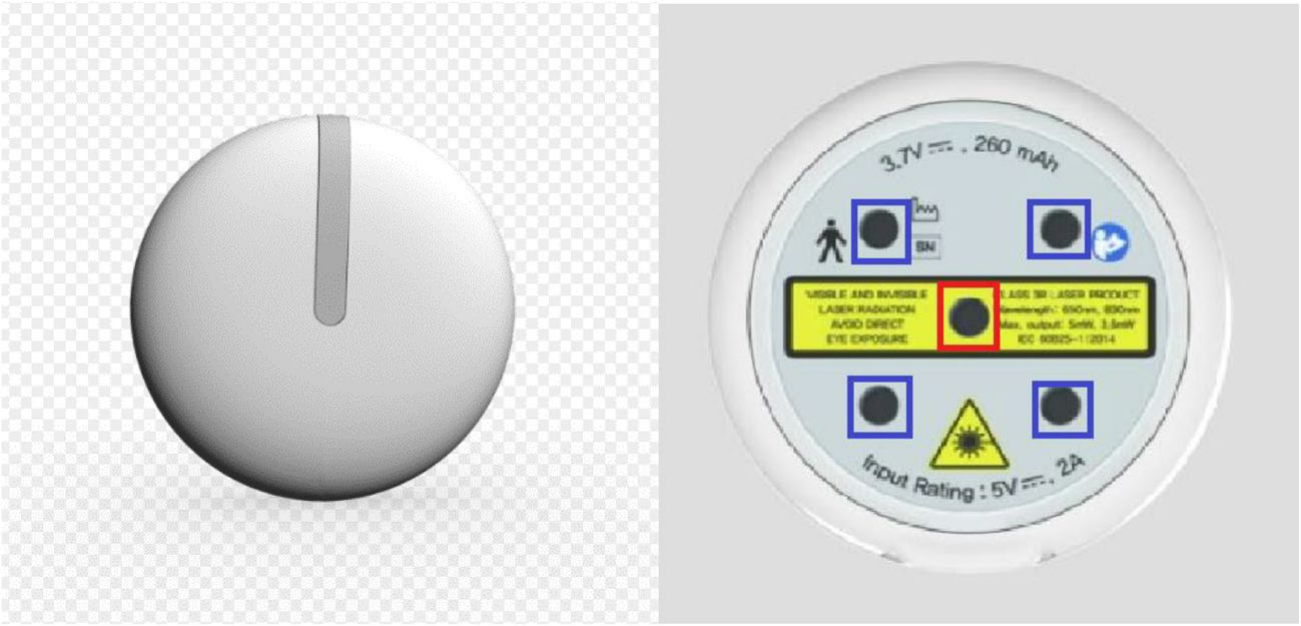
Epione™ (laser is generated from spots marked with red rectangle; a wavelength 830 nm at center, 630 nm at 4 peripheral area)

The device used in the current study is a small and attachable LLLT intended for home therapy. This device, with its directionality of the laser, is capable of transmitting predetermined amounts of energy to the precise area where pain relief is sought. Furthermore, patients can power the device on and off themselves and access user treatment guidelines through a mobile application linked to the device. A LLLT device is conducted using an effective complex wavelength spectrum (light sources in square configuration: 830 nm GaAs diode 3.2 mW at the center, 4 × 650 nm InGaAIP diodes over the corners. It emits continuous radiation from all of its apertures with an irradiance of 5.3 [mW/cm^2^] and a radiant exposure of 9.54 [J/cm^2^]. It is 48 mm diameter, 12 mm thickness, and 28 g weight) (Epione™, WellsCare Co., Seoul, Korea) (Fig. 1, Table 1). The experiment group received treatment with the stimulating laser. The control group was treated with a sham stimulation device that mimicked the shape and color of the Epione™ (sham stimulation radiated wavelength 830 nm GaAs diode 0.0002 mW at the center and wavelength 650 nm, average radiant power 0.025 mW at the corners).

**Table 1:** Dosimetric parameters of device

Parameter [unit]	Value	
Manufacturer	Well’s care	
Area	Center	Peripheral
Number of Emitters	1	4
Wavelength [nm]	830	650
Average radiant power [mW]	3.2	3.5
Transverse diameter [mm]	48	
Longitudinal diameter [mm]	48	
Thickness [mm]	12	
Weight [g]	28	
Beam area of the irradiation [cm^2^]	3.24	
Irradiance [mW/cm^2^]	5.3	
Exposure duration [second]	1800	
Radiant exposure [J/cm^2^]	9.54	
Total energy [J]	28.8	
Emitter type	Continuous emission	
Beam delivery system	Direct exposure	

### Outcome measures

Single-blinded evaluator who did not participate in the intervention performed all measurements. One evaluator performed the two evaluations on each subject.

#### Primary outcome

The primary outcome was assessed using a visual analogue scale (VAS) for wrist pain. The patient marked the degree of pain on a 100-mm line and measured the distance. A score of 0 indicated a lack of pain while a score of 10 indicated extreme pain [15].

#### Secondary outcome

Secondary outcomes were measured using a patient-rated wrist evaluation (PRWE) questionnaire, and by assessing grip strength, as well as lateral, palmar, and tip pinch strength.

The PRWE is a self-administered patient-specific questionnaire consisting of 15 items designed to measure wrist pain and disability in activities of daily living. It includes two subscales: pain and function. The pain subscale is comprised of five items, while the function subscale is divided between six specific activities and four typical activities. The pain subscale score represents the sum of the five associated items. The function subscale score is calculated by taking the sum of the ten related items and dividing by two. A patient’s PRWE score is the sum of those from each subscale. A score of 100 represents the worst functional score, whereas 0 represents no disability [16].

Hand grip strength was also used to assess muscle strength. A hand dynamometer (JAMAR®, Chicago, IL, USA) was used to measure maximum grip strength (Kg) in an elbow flexion and shoulder abduction position [17]. Pinch strength was measured for the three pinch types (tip, palmar, and lateral). Tip pinch, which measures the direct strength of the two fingers, was measured having subjects grip the objects with the tip of their index finger and the thumb. Palmar pinch was measured by having subjects grip the objects with their thumb, index finger, and middle finger. Lateral pinch was measured by gripping the objects with the thumb and the width during each test. The subject could start gripping or pinching with the same force using the same muscles [18]. Grip or pinch strength was measured on one hand to be treated, and the highest value was obtained by measuring the hand strength twice in each position used.

Safety was assessed by monitoring adverse reactions, including, checking vital signs, and through subject self-reporting of symptoms by patients.

### Sample size

A previous study concerning the therapeutic effects and neurological symptoms associated with low-level laser treatment in response to carpal tunnel syndrome used a Painless Light PL-830 (Advanced Chips & Products Corp., USA), which is similar to our tested device [5]. In that study, the expected average difference in visual analogue scale (VAS) score was − 0.276 and the standard deviation was 1.48 in the experiment group (*n* = 45). The control group (*n* = 42) showed average difference in VAS score was − 0.50 and the standard deviation was 0.83. The sample size necessary for our study was calculated to be 12, accounting for a drop rate of 20% by G*Power using the *t* test; the effect size 1.88, significance level 0.5, and the power 0.8 [19]. In fact, we ultimately enrolled 30 patients.

### Data analysis

Statistical analysis was performed using SPSS 23.0 software for Windows (SSPS Inc., Chicago, IL, USA). Data were presented as mean (SD) for continuous variables and frequency for categorical variables. For baseline demographic characteristics, the independent *t* test was used when the assumption to normality was satisfied and the Mann–Whitney *U* test was used otherwise. Fisher’s exact test was used to compare differences between groups for categorical variables. For comparisons between groups, the independent *t*-test was used when the assumption of normality was satisfied and the Mann–Whitney *U*-test was used otherwise. Within-group comparisons were assessed by paired *t*-test when the assumption of normality was satisfied and the Wilcoxon signed rank test was used otherwise. A *p* value ≤ 0.05 was considered statistically significant.

## Results

### Flow of participants, therapists, and centers through the study

**Fig. 2:**
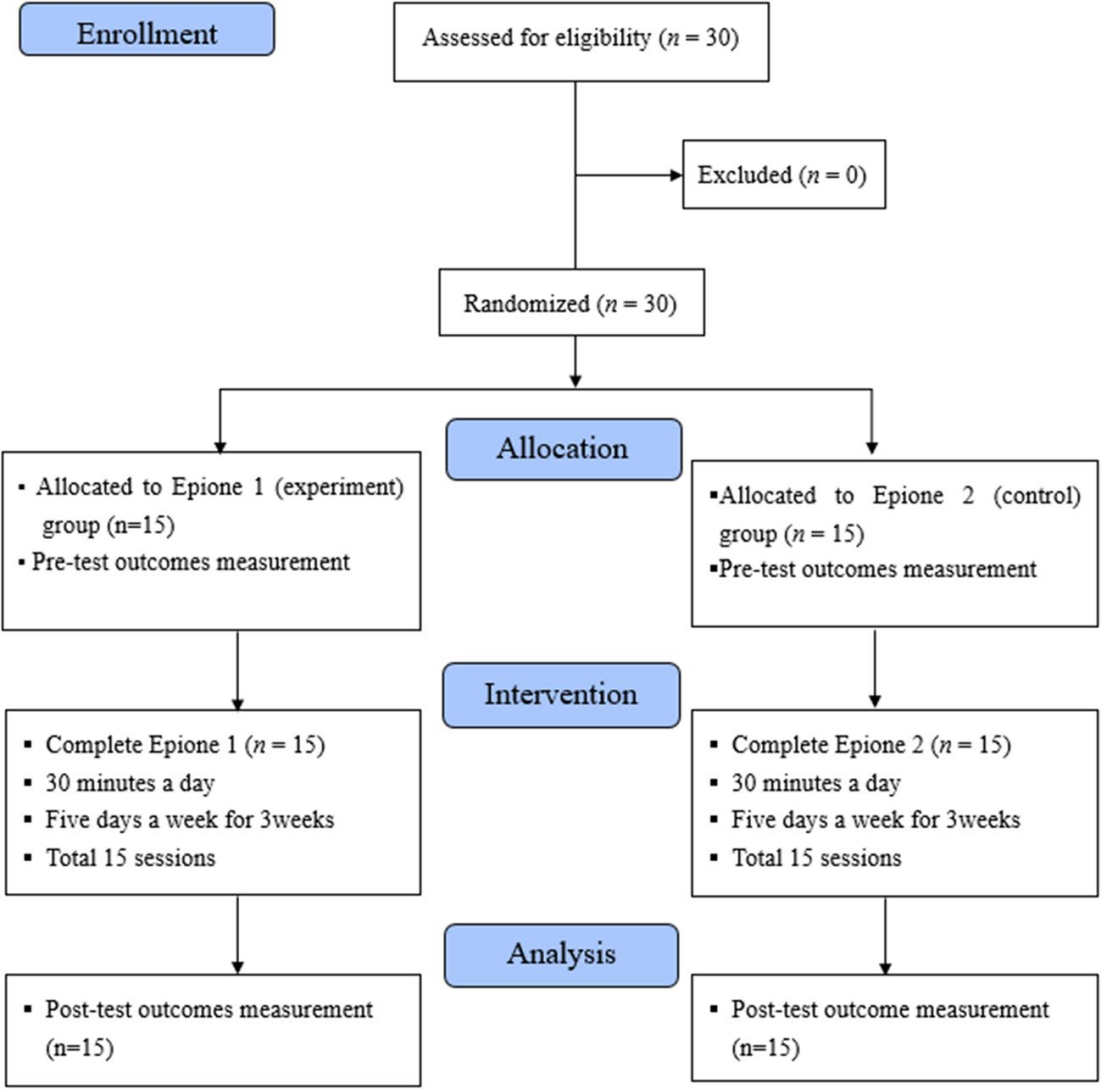
CONSORT flow diagram

Thirty patients recruited between 02 February 2021 and 31 July 2021 were randomly allocated to one of two groups, 15 in the experimental group and 15 in the control group. All participants completed treatment and evaluation (Fig. 2). Demographic data of age and sex showed no significant differences in baseline between the two groups.

### Effect of intervention

#### Primary outcomes

The results showed a significant improvement in VAS scores in both the experimental group (pretreatment: 4.93 ± 1.62, post-treatment: 3.67 ± 1.68, $\Delta$ 1.27 ± 1.87, *p* = 0.020) and the control group (pretreatment: 5.53 ± 1.36, post-treatment: 4.00 ± 2.00, $\Delta$ 1.53 ± 1.30, *p* = 0.003) in the within-group comparison. However, there were no significant differences in VAS score changes between the two groups. These results are presented in Table 2.

**Table 2:** Results of primary and secondary outcomes according to time and groups

		Experimental group (*n* = 15)				Control group (*n* = 15)				Between groups *p* value
		Pre treatment	Post-treatment	Post-treatment-Pretreatment	Within-group *p* value	Pre treatment	Post-treatment	Post-treatment-Pretreatment	Within-group *p* value	
VAS		4.93 ± 1.62	3.67 ± 1.68	1.27 ± 1.87	0.020*	5.53 ± 7.01	4.00 ± 2.00	1.53 ± 1.30	0.003*	0.225
PRWE	Pain	27.07 ± 7.57	21.07 ± 8.26	6.00 ± 7.41	0.007*	29.13 ± 7.01	20.20 ± 10.46	8.93 ± 5.38	< .001*	0.225
	Function	41.53 ± 20.88	28.80 ± 14.30	12.73 ± 17.20	0.012*	33.67 ± 20.46	26.27 ± 19.97	7.40 ± 17.14	0.117	0.085
	Total	68.60 ± 26.83	49.87 ± 21.38	18.73 ± 20.96	0.004*	62.80 ± 26.09	46.47 ± 29.56	16.33 ± 20.88	0.009*	0.406
Strength	Grip	23.02 ± 10.70	24.60 ± 10.80	1.58 ± 4.66	0.140	21.91 ± 7.16	23.25 ± 6.95	1.33 ± 3.18	0.127	0.867
	Pinch lateral	5.92 ± 2.12	6.53 ± 2.24	0.61 ± 0.78	0.017*	5.50 ± 1.66	5.82 ± 1.30	0.32 ± 1.34	0.365	0.198
	Pinch palmar	5.00 ± 1.97	5.45 ± 1.90	0.45 ± 0.86	0.083	4.82 ± 1.27	5.00 ± 1.17	0.18 ± 0.85	0.416	0.171
	Pinch tip	4.54 ± 2.01	4.97 ± 1.59	0.43 ± 0.97	0.088	4.07 ± 1.06	4.51 ± 1.26	0.44 ± 0.72	0.034*	0.992

^*^p < 0.05, *VAS* Visual Analogue Scale, *PRWE* patient rated wrist evaluation

#### Secondary outcomes

In the within-group comparison of PRWE scores, both groups showed significant improvement in pain subscale scores (*p* = 0.007 in experimental group, *p* ≤ 0.001 in control group) and total scores (*p* = 0.004 in experimental group, *p* = 0.009 in control group), while function subscale scores significantly improved only in the experimental group (*p* = 0.012). No significant differences are observed between the two groups (Table 2). Seventy-three percent of the experimental group patients (11/15) and 53% of the control group patients (8/15) reached the minimal clinical important difference (MCID).

In the within-group comparison, the lateral pinch strength results revealed a significant improvement (*p* = 0.017) in the experimental group, as well as a significant improvement in tip pinch strength (*p* = 0.034) in the control group. No other significant differences in results were observed in the in-group comparison. No significant differences are observed between the two groups (Table 2).

## Discussion

In this study, we investigated the effect of compact laser irradiation on individuals suffering from wrist pain. In the primary outcome, both groups showed significant improvements in their VAS pain scores, although there were no significant differences between the groups. As to the secondary outcomes, both groups showed significantly improved pain subscale and total scores on the PRWE evaluation, with the experimental group reporting significant improvement in the function subscale and lateral pinch strength, and the control group reporting significant increase in tip pinch strength. There were no significant differences between the control and experiment groups in the secondary outcomes.

Several pre-clinical studies have reported on the results of using PBMT to treat various conditions, including arthritis, tendinitis, and inflammation [20]. A previous in vivo study demonstrated the efficacy of PBMT in controlling inflammatory mediators in rats with a chronic constriction of the sciatic nerve [21]. Another study reported the use of PBMT to promote analgesia and output, resulting in increased expression of β-endorphin and a significant reduction in pain [22]. PBMT is based on the principles of photochemistry that facilitated by exerting photochemical or non-thermal effects on cells. The treatment minimizes inflammation and edema at affected sites by stimulating microcirculation and by activating the terminal enzyme, cytochrome c oxidase [23–25]. The treatment has also been shown to promote wound healing in superficial and deep tissue layers, and help patients recover from neurological damage [24].

PBMT offers a non-invasive, safe, and side effect–free method for pain relief of both acute and chronic musculoskeletal conditions [26–28]. Also, there were many specific studies targeting patients complaining of wrist pain. In these studies, it was reported that PBMT showed significant improvement of several functional score including pain, PRWE, and grip strength, as well as enhanced bone healing after distal radius fracture and mild to moderate carpal tunnel syndrome [29–32].

In contrast, other studies have compared PBMT with ultrasound therapy or high-intensity laser therapy and concluded that PBMT offers no therapeutic benefit [33, 34]. Most of these studies have been conducted in hospitals because of large and heavy device. On the other hand, our device is light and portable, so we attempted to see the therapeutic efficacy and safety of the patients with wrist pain through home-based self-therapy, and the results of this study were obtained.

Our study showed a significant improvement in VAS and pain subscale scores in both the experiment and control groups. Notably, there was no significant difference between groups. All study participants initially complained of moderate wrist pain, which implied mild to moderate interference with functioning [35]. Perhaps the improvement in the control group is attributable to the performance of fewer daily living activities or a reduction in hand use during the treatment period. That similar results have been reported across several previous studies suggesting that further studies on PBMT are needed [36].

We observed a significant improvement in the total PRWE subscale scores in both the experiment and the control groups and function PRWE subscale scores only in the experimental group. WMSD patients often complain of pain and swelling, which is inconvenient and impairs hand function [37]. Reductions in wrist pain typically lead to functional improvements in the hand [36]. In our study, the experimental group reported a significant decrease in pain and a corresponding improvement to function. In the control group, no placebo effect led to functional improvement. This result is consistent with other studies which have found significant improvement to hand function post-PBMT, and suggests the therapeutic potential of the treatment [11, 36]. Also, we calculated MCID for PRWE. According to previous studies, the MCID of PRWE is 14 [38]. When the MCID for PRWE was calculated, both groups showed improvement compared before and after treatment, but the result was less than the MCID.

Our participants in the experiment group reported significant improvement in lateral pinch strength. The control group reported a significant improvement in tip pinch strength, though there was no statistical difference between groups. We hypothesize that the effect of treatment on lateral, palmar, and tip pinch strength is different depending on the location of wrist pain. For example, patients with DeQuervain syndrome, who mainly complain of radial side pain, could show the impairment of lateral pinch strength [39]. As our study participants were not limited to a specific disease group (e.g., DeQuervain syndrome, carpal tunnel syndrome, or osteoarthritis), this variability may account for the grip and pinch strength results.

This study had several limitations. First, participants were not confined to a particular wrist disease group and as a result the possibility exists that the response to treatment was inconsistent. Second, our study is lack of analysis of various treatment parameters (i.e., laser wavelength and treatment intensity). Third, our study consisted of only a small number of participants.

In conclusion, as more people use computers and smartphones for extended periods and the incidence of work-related musculoskeletal disorders increases, more accessible and portable treatment device for associated conditions will be needed. This medical laser irradiation is a portable and easy-to-use laser irradiators without side effects. Furthermore, more research is still needed on physical therapeutic modality that is easily accessible and more portable for musculoskeletal pain patients.

## Data Availability

The original contributions presented in the study are included in the article; further inquiries can be directed to the corresponding author.
